# Association of alanine aminotransferase concentration with cardiometabolic risk factors in children and adolescents: the CASPIAN-V cross-sectional study

**DOI:** 10.1590/1516-3180.2018.0161161118

**Published:** 2018-12-13

**Authors:** Roya Kelishadi, Mostafa Qorbani, Ramin Heshmat, Nazgol Motamed-Gorji, Mohammad Esmaeil Motlagh, Hasan Ziaodini, Majzoubeh Taheri, Gita Shafiee, Tahereh Aminaee, Zeinab Ahadi, Motahar Heidari-Beni

**Affiliations:** I MD. Professor, Department of Pediatrics, Child Growth and Development Research Center, Research Institute for Primordial Prevention of Noncommunicable Disease, Isfahan University of Medical Sciences, Isfahan, Iran.; II PhD. Epidemiologist andAssistant Professor,Non-communicable Diseases Research Center, Alborz University of Medical Sciences, Karaj, Iran; and Chronic Diseases Research Center, Endocrinology and Metabolism Population Sciences Institute, Tehran University of Medical Sciences, Tehran, Iran.; III MD. Associate Professor, Chronic Diseases Research Center, Endocrinology and Metabolism Population Sciences Institute, Tehran University of Medical Sciences, Tehran, Iran.; IV MD.Researcher,Chronic Diseases Research Center, Endocrinology and Metabolism Population Sciences Institute, Tehran University of Medical Sciences, Tehran, Iran.; V MD. Professor, Department of Pediatrics, Ahvaz Jundishapur University of Medical Sciences, Ahvaz, Iran.; VI MD. Researcher, Office of Health and Fitness, Ministry of Education, Tehran, Iran.; VII MD. Pediatrician and Researcher, Bureau of Population, Family and School Health, Ministry of Health and Medical Education, Tehran, Iran.; VIII MD. Researcher, Chronic Diseases Research Center, Endocrinology and Metabolism Population Sciences Institute, Tehran University of Medical Sciences, Tehran, Iran.; IX BSc. Researcher, Bureau of Population, Family and School Health, Ministry of Health and Medical Education, Tehran, Iran.; X MSc. Researcher, Chronic Diseases Research Center, Endocrinology and Metabolism Population Sciences Institute, Tehran University of Medical Sciences, Tehran, Iran.; XI PhD. Nutritionist and Assistant Professor, Department of Pediatrics, Child Growth and Development Research Center, Research Institute for Primordial Prevention of Noncommunicable Disease, Isfahan University of Medical Sciences, Isfahan, Iran.

**Keywords:** aminotransferase, Risk factors, Child

## Abstract

**BACKGROUND:**

It has been suggested that the levels of some liver enzymes, and especially alanine aminotransferase (ALT), might be correlatable with cardiometabolic risk factors. We investigated the relationship between ALT concentration and cardiometabolic risk factors among children and adolescents.

**DESIGN AND SETTING:**

This nationwide study in Iran was conducted within the framework of the fifth survey of a national surveillance program known as the Childhood and Adolescence Surveillance and PreventIon of Adult Non-communicable disease study (CASPIAN-V).

**METHODS:**

The participants comprised 4200 students aged 7-18 years, who were recruited through multi-stage random cluster sampling in 30 provinces in Iran. Physical examinations and laboratory tests were conducted in accordance with standard protocols.

**RESULTS:**

Overall, 3843 students (participation rate: 91.5%) completed the survey. Mean ALT levels were significantly higher in individuals with dyslipidemia, in terms of elevated total cholesterol (TC) or LDL-cholesterol or triglycerides (TG), excess weight and dyslipidemia. Some cardiometabolic risk factors were associated with higher levels of ALT, with the following odds ratio (OR) and 95% confidence interval (CI):metabolic syndrome (OR: 1.013; 95% CI: 1.001-1.025); elevated TC (OR: 1.060; 95% CI: 1.039-1.081), elevated LDL (OR: 1.031; 95% CI: 1.016-1.046), elevated TG (OR: 1.056; 95% CI: 1.040-1.072) and dyslipidemia (OR: 1.051; 95% CI: 1.034-1.068).

**CONCLUSION:**

This large population-based study revealed that some cardiometabolic risk factors were significantly associated with ALT levels. These findings suggest that an association with fatty liver is an underlying mechanism for development of cardiometabolic risk factors.

## INTRODUCTION

Nonalcoholic fatty liver disease (NAFLD) is the most prevalent liver disorder. Recently, an association between NAFLD and metabolic syndrome (MetS) has been demonstrated. However, it is unclear whether NAFLD causes metabolic dysfunction, or whether metabolic disorders are responsible for fatty liver disease, or whether both of these might occur.^1^ Cardiovascular diseases (CVDs) lead to death among individuals with NAFLD. However, it is less clear whether NAFLD increases morbidity or mortality relating to CVD.^2^

Several methods are used to diagnose NAFLD. Although liver biopsy is the standard for diagnosing of hepatic steatosis, ultrasonography and biochemical tests are easy to use in clinical plans. Serum biomarkers, particularly alanine aminotransferase (ALT) levels, are sensitive for detecting NAFLD in both obese and non-obese patients.^3^ Because ALT is closely related to liver fat accumulation, ALT is used as a surrogate marker for NAFLD in epidemiological studies.^4^

It has been suggested that serum ALT is the liver enzyme most closely correlated with liver fat content. Elevated ALT levels in cases of obesity have physiological significance in terms of the potential effect of fatty liver, which commonly occurs in cases of MetS. Thus, it has been shown that elevated ALT levels are associated with the incidences of MetS, diabetes mellitus^5^^,^^6^ and cardiovascular disease, independently of traditional risk factors.^7^

In addition, correlations between ALT levels and both waist circumference and higher circulating insulin levels have been found. Hepatic insulin resistance leads to subsequent decline in hepatic insulin sensitivity.^8^^,^^9^ Recent studies have indicated that higher levels of abdominal fat, particularly visceral fat, are closely correlated with occurrences of NAFLD. However, data on the relationship between ALT concentrations, visceral fat accumulation and cardiometabolic risk factors remain rare. These associations are considerable and need to be studied further.^10^^,^^11^

The Framingham Heart Study showed that ALT levels were associated with multiple cardiometabolic risk factors.^6^In the Korean National Health and Nutrition Examination Surveys, an association between increasing ALT levels and the presence of MetS was found.^12^ In addition, a cross-sectional study on a rural Chinese population showed that increased ALT levels were correlated with a worse cardiometabolic risk profile.^13^ A study on 5,586 adolescents reported that a relationship existed between ALT levels and waist circumference and insulin levels.^14^ An independent correlation between elevated liver enzyme levels and adverse CVD events was demonstrated in a meta-analysis on 10 pooled studies.^15^ These findings confirmed the potential for ALT levels to act as a biomarker for the risk of metabolic disease. Recently, NAFLD has come to be considered to be a new and important cardiovascular risk factor. Insulin resistance, obesity and increased triglyceride levels are important determinants of NAFLD and lead to increased risk of CVD.^16^

The relationship between ALT levels and some cardiometabolic risk factors among children and adolescents is unclear. To investigate this hypothesis, we studied the relationship between ALT levels and risk factors for MetS and CVD in the Childhood and Adolescence Surveillance and PreventIon of AdultNoncommunicable disease (CASPIAN-V) study.

## METHODS

The data for this study were collected as part of the “National Survey of School Student High-Risk Behaviors” (2014-2015). Thisconstituted the fifth survey of the school-based surveillance system, known as the Childhood and Adolescence Surveillance and PreventIon of Adult Noncommunicable disease (CASPIAN-V) study. This school-based nationwide health survey was conducted in 30 provinces in Iran. Details of the study protocol have been discussed previously,^17^ and are reported here briefly as well.

### Study population and sampling

The study population consisted of students aged 7-18 years in primary and secondary schools in urban and rural areas across the country. They were selected using a multistage stratified cluster sampling method. Sampling within each province was conducted according to the student’s place of residence (urban or rural) and level of education (primary and secondary) using the proportional-to-size method and with a 1:1 sex ratio.

The desired number of samples was achieved using cluster sampling in each province, with equal cluster sizes. This was a multistage stratified cluster sampling method in which the clusters were determined at school level. The size of each cluster was 10students, meaning that a total of 10 sampling units (including 10 students and their parents) would be considered in each cluster. The sample size of the main survey included 480 students in each province (48clusters of 10 students), i.e. a total of 14,400 students at national level. In each province, 14 out of the 48 clusters were randomly selected for biochemical tests. Therefore, the sample size of the current study was estimated to be 4200.

### Procedures and measurements

#### Questionnaires

Two questionnaires were used: one for students and the other for their parents. The students’ questionnaire was derived from the World Health Organization Global School Student Health Survey (WHO-GSHS) (Persian-translated version). The validity and reliability of this questionnaires had been assessed previously.^18^^,^^19^ An expert panel approved the validity and, in the phase of content validity assessment, questions with a score of more than 0.75 were approved as having optimal content validity.

After eligible students had been identified, the mission and purpose of the interview was explained to them. They were told that the questions were about the health status and health-related behaviors of children and adolescents. Interviews were conducted in a peaceful environment. The questions were read out to the students using simple words, and they could not see the questions. The whole process was supervised and controlled by a team of healthcare professionals. This questionnaire included questions about physical activity status and screen time status.

After the eligible students had been identified, their parents were invited to complete the parents’ questionnaire. The presence of at least one of the parents was necessary and sufficient. The parents were informed that the questions were about health status and health-related behaviors in the students’ families. Theparents’ questionnaire also sought information on family characteristics, namely household size, number of students and socioeconomic variables.

#### Physical measurements

A team of trained healthcare experts recorded information. Theyperformed the examinations in accordance with standard protocols and used calibrated instruments. Weight was measured to the nearest 0.1 kg on a scale placed on a level floor, while the subjects were wearing light clothes; and height were measured to the nearest 0.1 cm, without shoes.^19^ Body mass index (BMI) was calculated by dividing weight (kg) by height squared (m^2^). Weused the WHO growth charts to categorize BMI.^20^

Waist circumference was measured to the nearest 0.1 cm using a non-elastic tape, at a point midway between the lower border of the rib cage and the iliac crest at the end of normal expiration. Hipcircumference was measured to the nearest 0.1 cm, at the widest part of the hip at the level of the greater trochanter.^21^ Wrist circumference was measured to the nearest 0.1 cm on the dominant arm using a non-elastic tape. The subjects were asked to rest their arm on a flat surface, such as a table. The upper edge of the tape measure was placed just distally to the prominences of the radial and ulnar bones. Neck circumference was measured to an accuracy of 0.1 cm, taking the most prominent portion of the thyroid cartilage as a landmark.

Blood pressure was measured in the sitting position on the right arm using a mercury sphygmomanometer with an appropriate cuff size. It was measured twice, with a five-minute interval between the measurements. Systolic and diastolic pressures were recorded and the average was registered.^22^

#### Laboratory sampling

Selected students were referred to the laboratory (i.e. the laboratory that was the nearest to the school) for blood sampling, and one of each student’s parents accompanied him/her. A sample of 6 ml of venous blood was collected after 12-hour overnight fasting. All collection tubes were centrifuged at 2500-3000 x g for 10 minutes. Immediately after centrifugation, the serum samples were aliquoted into 200-microliter tubes and were stored at-70°C. All the samples were transferred by means of a cold chain to Isfahan Mahdieh Laboratory. A cold chain is a system used for keeping biological samples in good conditions and this system consists of a series of links that are designed to keep biological samples within the temperatures ranges recommended by the World Health Organization, from the point of manufacture to the point of administration. One common temperature range for a cold chain is 2 to 8°C. Alanine aminotransferase (ALT), fasting blood glucose (FBG), triglycerides (TG), total cholesterol (TC), low-density lipoprotein-cholesterol (LDL-C) and high-density lipoprotein-cholesterol (HDL-C) were measured enzymatically using a Hitachi auto-analyzer (Tokyo, Japan).^23^^,^^24^

### Definitions

WHO growth curves were used to define age and sex-specific BMI categories. Underweight was defined as BMI < 5^th^ percentile, overweight as BMI as 85^th^-95^th^, excess weight as BMI > 85^th^ and generalized obesity as > 95^th^. On the other hand, abdominal obesity was defined as waist-to-height ratio (WHtR) greater than or equal to 0.5.^25^

ALT > 35 IU/l was considered high. Fasting blood glucose (FBG) ≥ 100 mg/dl, serum triglycerides (TG) ≥ 100 mg/dl, total cholesterol (TC) ≥ 200 mg/dl, LDL-C ≥ 110 mg/dl and HDL-C <40 mg/dl (except for boys aged 15-18 years, for whom it was <45mg/dl) were considered abnormal.

Elevated blood pressure was defined as either high systolic blood pressure (SBP) or high diastolic blood pressure (DBP)≥90^th^ percentile for age, sex and height. Metabolic syndrome was defined in accordance with the modified NCEP ATP III criteria.^26^

MetS was defined as clustering of at least three of the five cardiometabolic risk factors, including abdominal (central) obesity, elevated BP, elevated fasting plasma glucose (FPG), high serum TG and low serum HDL. WHtR > 0.5 was considered to represent abdominal obesity. Dyslipidemia was determined as the presence of at least one abnormal lipid profile component (i.e. TG, TC, HDL or LDL).

### Physical activity (PA)

It was considered that the students were doing enough PA if they did exercises lasting at least 30 minutes per day (theresponse options ranged from 0 to 7 days) that led to sweating and large increases in respiratory or heart rate.^27^ Evaluation of PA was done via two questions: 1) “On how many days were you physically active for an overall 30 minutes per day during the past week?”; and 2) “On a regular basis, how much time do you spend in physical education (PE) classes at school per week?”. PA of less than 2 hours per week was considered low, while 2-4hours a week was considered moderate and more than 4hours a week was considered high.

### Screen time (ST)

To assess ST behavior, the students were asked how many hours per day they were spending watching television and/or videos, using a personal computer or playing electronic games. Fromthis, the total cumulative time spent as ST was calculated.

### Socioeconomic status (SES)

SES scores were estimated using the principle component analysis (PCA) method, based on parents’ education and job, type of school (private or governmental), type of home (private/rented) and family assets (private car and computer). The SES score for each student was a weighted average of the SES variables. The weighted averages of these variables were summarized under one main component named SES score. Students were classified as having low, moderate or high socioeconomic status, based on this component.

### Ethical concerns

The study protocols were reviewed and approved by the ethics committees of Isfahan University of Medical Sciences. TheResearch Ethics Council of Isfahan University of Medical Sciences approved the study (project number: 194049). Aftercomplete explanation of the study objectives and protocols, written informed consent and verbal consent were obtained from the parents and students, respectively.

### Statistical analysis

The data were expressed as means and standard deviations (SD) for continuous variables, and as numbers (percentages) for categorical variables. ALT data were transformed to normalize their skewed distribution. The Student t test was used to compare variables with two groups. The ANOVA test was used to compare means for variables with more than two groups. Associationsbetween qualitative variables were assessed using the Pearson chi-square test. Back transformation was applied to the final findings.

Logistic regression analysis was performed to evaluate associations between ALT and abnormal cardiometabolic outcomes (presence of high BP, high fasting blood sugar (FBS), high TG, high TC, high LDL, low HDL, overweight, obesity, excess weight, abdominal obesity, MetS and dyslipidemia). Two models were defined for each association: model I represented the crude association; and model II was adjusted for age, sex, ST, PA, SES and living area. The results from the logistic regression were represented as odds ratios (OR) and 95% confidence intervals (CI).

Linear regression analysis was conducted to evaluate associations between ALT and the quantitative values of cardiometabolic risk factors, including BMI, waist circumference, neck circumference, wrist circumference, hip circumference, SBP, DBP, HDL, LDL, TG, TC and FPG. The data were displayed via the β-coefficient and standard error (SE). Again, two models were defined for each association: model I represented the crude association; and model II described the model I associations after adjustment for age, sex, ST, PA, SES and living area.

A P-value of < 0.05 was considered statistically significant in all measurements. All statistical measurements were estimated using survey data analysis methods. The data were analyzed by using the STATA package, version 11.0 (Stata Statistical Software: Release 11; Stata Corp LP, College Station, TX, USA).

## RESULTS

Overall, 3,843 students (out of 4,200 students who had been selected) completed the survey (response rate: 91.5%). Thegeneral characteristics of the participants are presented in Tables1and2. Overall, the mean age of the students was 12.58years (±3.15). 52.6% of the sample consisted of boys, and 72.7% lived in urban regions.

Nearly 60% of the students had dyslipidemia, although the majority of the students affected by this only had one abnormal component (TG, HDL, LDL or TC). Low HDL was seen in approximately 30% of the students. Overall, 20% of the subjects presented excess weight (BMI > 85^th^ percentile), and 10% had high blood pressure (either systolic or diastolic, or both). High ALT was seen in only 13 students (0.3%).

**Table 1.** Participants’ demographic characteristics in numbers and percentages, according to presence of metabolic syndrome: the CASPIAN-V study

Variables^*^		Overall (total = 3,731) n (%)	MetS (total = 188) n (%)
Gender	Boys	1,964 (52.6)	108 (57.4)
	Girls	1,768 (47.4)	80 (42.6)
Region	Urban	2,714 (72.7)	154 (81.9)
	Rural	1,018 (27.3)	34 (18.1)
Dyslipidemia		2,085 (55.9)	187 (99.5)
Dyslipidemia components	0	1,647 (44.1)	1 (0.5)
	1	1,353 (36.3)	53 (28.2)
	2	590 (15.8)	108 (57.4)
	3	122 (3.3)	20 (10.6)
	4	20 (0.5)	6 (3.2)
Abdominal obesity		756 (20.3)	133 (70.7)
Generalized obesity		408 (10.9)	68 (36.2)
Excess weight		747 (20.0)	96 (51.1)
High systolic BP		93 (2.5)	20 (10.6)
High diastolic BP		345 (9.2)	81 (43.1)
High BP		374 (10.0)	84 (44.7)
High TC, mg/dl		185 (5.0)	9 (4.8)
Low HDL-C, mg/dl		1,106 (29.6)	154 (81.9)
High LDL-C, mg/dl		659 (17.7)	30 (16.0)
High TG, mg/dl		1,029 (27.6)	160 (85.1)
High FBG, mg/dl		158 (4.2)	47 (25.0)
High ALT		13 (0.3)	3 (1.6)

^*^Data are presented as number (percentage). MetS = metabolic syndrome (defined according to ATP-III criteria); SBP=systolic blood pressure; DBP = diastolic blood pressure; BP=blood pressure; TC = total cholesterol; HDL = high density lipoprotein; LDL=low density lipoprotein; TG = triglycerides; FBG=fasting blood glucose; ALT=alanine aminotransferase; dyslipidemia: at least one of the components (TG, HDL, LDL or TC) was abnormal; excess weight: BMI>85^th^ percentile; obesity, BMI > 95^th^ percentile; low HDL: < 40­mg/­dl (except among boys aged 15-19years, for whom the cutoff was <45­mg/­dl); high LDL: > 110­mg/­dl; highTG:100 mg/dl; high TC: > 200­mg/­dl; high FBS > 100 mg/dl; high blood pressure: > 90^th^ percentile (adjusted according to age, sex and height); high ALT: > 35 IU/l.

**Table 2.** Participants’ demographic characteristics in means and standard deviations, according to presence of metabolic syndrome: the CASPIAN-V study

Variables^*^	Overall (total = 3,731) mean (SD)	MetS (total = 188) mean (SD)	Non-MetS (total = 3,543) mean (SD)	P-value
Age, years	12.283 (3.158)	12.409 (2.893)	12.443 (3.046)	0.882
BMI, kg/m^2^	18.510 (4.713)	21.998 (9.754)	18.296 (4.197)	< 0.001
Waist circumference, cm	66.723 (12.177)	76.704 (13.995)	66.160 (11.658)	< 0.001
Hip circumference, cm	79.144 (14.642)	86.556 (15.455)	79.142 (14.342)	< 0.001
Neck circumference, cm	29.845 (3.999)	31.650 (4.057)	29.860 (3.909)	< 0.001
Wrist circumference, cm	14.722 (1.891)	15.800 (1.985)	14.713 (1.783)	< 0.001
ALT IU/l	8.332 (7.071)	9.760 (7.309)	8.288 (7.141)	0.008
SBP, mmHg	99.170 (13.095)	106.143 (14.401)	98.323 (12.711)	< 0.001
DBP, mmHg	63.836 (10.435)	71.531 (11.773)	63.122 (9.931)	< 0.001
TC, mg/dl	153.85 (27.424)	152.780 (27.231)	153.94 (27.504)	0.573
HDL-C, mg/dl	46.190 (9.976)	37.210 (7.259)	46.64 (9.922)	< 0.001
LDL-C, mg/dl	90.052 (22.609)	87.145 (22.800)	90.249 (22.656)	0.067
TG, mg/dl	88.04 (45.180)	142.14 (61.844)	85.23 (42.656)	< 0.001
FBG, mg/dl	91.65 (12.111)	98.34 (14.861)	91.29 (11.899)	< 0.001

^*^Data are presented as mean ± standard deviation. MetS = metabolic syndrome (defined according to ATP-III criteria); ALT=alanine aminotransferase; SBP =systolic blood pressure; DBP=diastolic blood pressure; TC = total cholesterol; HDL = high density lipoprotein; LDL = low density lipoprotein; TG = triglycerides; FBG = fasting blood glucose.

Overall, 188 students out of 3731 (5.039%) were classified as having MetS. In comparing cardiometabolic risk factors between the groups with and without MetS, most of the cardiometabolic risk factors were significantly more prevalent in the MetS group, including abdominal obesity (70.7%), generalized obesity (36.2%), high BP (44.7%), low HDL (81.9%), high TG (85.1%), high FBG (25%) and high ALT (1.6%) (Table 1). Table 2 displays the continuous values for cardiometabolic outcomes according to MetS status. Overall, the MetS and non-MetS groups showed significant differences in almost all risk factors (except for TC and LDL), thus suggesting MetS is associated with higher values for cardiometabolic risk factors (Table 2).

The mean ALT levels according to the cardiometabolic risk factors and characteristics of the study population are demonstrated inTable3. These levels were significantly higher in individuals with high TC, high LDL, high TG, excess weight and dyslipidemia. Themean ALT level increased monotonically with increasing numbers of MetS components and with increasing dyslipidemia (Table 3).

**Table 3.** Mean alanine aminotransferase (ALT) levels according to cardiometabolic risk factors: the CASPIAN-V study

Outcomes		ALT		
		Mean (IU/l)	SD	P-value
Gender	Boy	8.49	5.11	0.14
	Girl	8.15	8.73	
Age	7-10	8.43	6.77	0.83
	11-14	8.26	4.79	
	15-18	8.33	9.88	
Region	Urban	8.41	7.9	0.13
	Rural	8.11	4.16	
Physical activity	High	8.42	5.37	0.52
	Low	8.27	8.09	
Socioeconomic status	High	8.15	5.29	0.77
	Medium	8.37	7.42	
	Low	8.21	5.74	
High SBP	Yes	8.244	6.337	0.879
	No	8.358	7.150	
High DBP	Yes	7.971	4.972	0.290
	No	8.395	7.318	
High TC	Yes	13.052	23.302	0.004
	No	8.088	4.840	
Low HDL-C	Yes	8.586	10.755	0.280
	No	8.226	4.747	
High LDL-C	Yes	9.623	8.522	< 0.001
	No	8.058	6.691	
High TG	Yes	9.590	10.369	< 0.001
	No	7.850	5.210	
High FBG	Yes	8.422	4.738	0.869
	No	8.328	7.156	
Abdominal obesity	Yes	8.866	7.721	0.250
	No	8.220	6.933	
Generalized obesity	Yes	8.920	6.422	0.081
	No	8.277	7.175	
Excess weight	Yes	8.808	7.965	0.045
	No	8.231	6.861	
Dyslipidemia	Yes	8.848	8.806	< 0.001
	No	7.684	3.828	
Number of MetS components	0	7.856	4.301	< 0.001
	1	8.300	5.167	
	2	9.104	12.550	
	≥ 3	9.402	6.623	
Number of dyslipidemia components	0	7.684	3.828	< 0.001
	1	8.125	4.882	
	2	9.239	7.164	
	≥ 3	14.11	7.755	

MetS = metabolic syndrome (defined according to ATP-III criteria); SBP =systolic blood pressure; DBP = diastolic blood pressure; BP = blood pressure; TC = total cholesterol; HDL = high density lipoprotein; LDL = low density lipoprotein; TG= triglycerides; FBG = fasting blood glucose; ALT = alanine aminotransferase; dyslipidemia: at least one of the components (TG, HDL, LDL or TC) was abnormal; excess weight: BMI > 85^th^ percentile; obesity, BMI > 95^th^ percentile; low HDL: <40­mg/­dl (except among boys aged 15-19 years, for whom the cutoff was <45mg/dl); high LDL: > 110 mg/dl; high TG: 100 mg/dl; high TC: > 200 mg/dl; high FBS > 100 mg/dl; high blood pressure: > 90^th^ percentile (adjusted according to age, sex and height). The Student t test was used to compare variables with two groups. The analysis of variance (ANOVA) test was used to compare means for variables with more than two groups.

Table 4 shows the associations between ALT levels and cardiometabolic risk factors in two different logistic regression models. Presence of MetS (OR: 1.013; 95% CI: 1.001-1.025), high TC (OR: 1.060; 95% CI: 1.039-1.081), high LDL (OR: 1.031; 95% CI:1.016-1.046), high TG (OR: 1.056; 95% CI: 1.040-1.072) and dyslipidemia (OR: 1.051; 95% CI: 1.034-1.068) were significantly associated with high ALT levels (Table 4). Table 5 presents the associations between ALT levels and cardiometabolic risk factors in linear regression models. In this analysis, high BMI (β: 0.013; SE: 0.010), high DBP (β: -0.056; SE: 0.023), high TC (β: 0.471; SE:0.064), high LDL (β:0.282; SE: 0.053) and high TG (β: 1.125; SE: 0.102) were significantly associated with high ALT levels (Table 5).

**Table 4.** Association of alanine aminotransferase (ALT) with cardiometabolic risk factors in logistic regression models: the CASPIAN V study

Outcomes	Model I^a^			Model II^b^		
	OR	95% CI	P-value	OR	95% CI	P-value
MetS	1.013	1.001-1.025	0.029	1.013	1.001-1.025	0.033
High BP	0.990	0.969-1.011	0.337	0.982	0.958-1.007	0.165
High SBP	0.997	0.965-1.031	0.878	0.985	0.939-1.034	0.543
High DBP	0.987	0.964-1.010	0.261	0.982	0.965-1.008	0.170
High TC	1.062	1.042-1.082	< 0.001	1.060	1.039-1.081	< 0.001
Low HDL-C	1.007	0.997-1.016	0.173	1.009	0.998-1.019	0.102
High LDL-C	1.032	1.017-1.047	< 0.001	1.031	1.016-1.046	< 0.001
High TG	1.051	1.036-1.066	< 0.001	1.056	1.040-1.072	< 0.001
High FBG	1.002	0.982-1.022	0.869	1.00	0.977-1.024	0.983
Abdominal obesity	1.011	1.001-1.021	0.049	1.009	0.999-1.020	0.079
Generalized obesity	1.009	0.998-1.020	0.111	1.007	0.996-1.019	0.208
Excess weight	1.009	0.999-1.020	0.072	1.007	0.997-1.017	0.158
Dyslipidemia	1.045	1.029-1.060	< 0.001	1.051	1.034-1.068	< 0.001

^a^Crude model; ^b^Adjusted for age, sex, PA, ST, SES and living area. MetS = metabolic syndrome (defined according to ATP-III criteria); SBP =systolic blood pressure; DBP = diastolic blood pressure; BP = blood pressure; TC = total cholesterol; HDL = high density lipoprotein; LDL = low density lipoprotein; TG=triglycerides; FBG = fasting blood glucose; ALT = alanine aminotransferase; dyslipidemia: at least one of the components (TG, HDL, LDL or TC) was abnormal; excess weight: BMI > 85^th^ percentile; obesity, BMI > 95^th^ percentile; low HDL: <40mg/dl (except among boys aged 15-19 years, for whom the cutoff was < 45mg/dl); high LDL: > 110 mg/dl; high TG: 100 mg/dl; high TC: > 200 mg/dl; high FBS > 100 mg/dl; high blood pressure: > 90^th^ percentile (adjusted according to age, sex and height).

**Table 5.** Association of alanine aminotransferase (ALT) levels with cardiometabolic risk factors in linear regression models: the CASPIAN V study

Outcomes	Model I^a^			Model II ^b^		
	β	SE	P-value	β	SE	P-value
BMI, kg/m^2^	0.015	0.011	0.149	0.013	0.010	< 0.001
Waist circumference, cm	0.059	0.028	0.033	0.044	0.025	0.070
Wrist circumference, cm	0.006	0.004	0.123	0.005	0.004	0.139
Hip circumference, cm	0.065	0.034	0.054	0.049	0.028	0.082
Neck circumference, cm	0.011	0.009	0.250	0.006	0.008	0.464
SBP, mmHg	-0.029	0.030	0.323	-0.043	0.028	0.124
DBP, mmHg	-0.052	0.023	0.026	-0.056	0.023	0.015
TC, mg/dl	0.481	0.062	< 0.001	0.471	0.064	< 0.001
HDL-C, mg/dl	-0.026	0.023	0.246	-0.036	0.023	0.122
LDL-C, mg/dl	0.284	0.051	< 0.001	0.282	0.053	< 0.001
TG, mg/dl	1.119	0.102	< 0.001	1.125	0.102	< 0.001
FBG, mg/dl	0.013	0.028	0.633	0.008	0.029	0.770

^a^Crude model; ^b^Adjusted for age, sex, PA, ST, SES and living area. MetS = metabolic syndrome (defined according to ATP-III criteria); ALT=alanine aminotransferase; SBP =systolic blood pressure; DBP=diastolic blood pressure; TC = total cholesterol; HDL = high density lipoprotein; LDL = low density lipoprotein; TG = triglycerides; FBG = fasting blood glucose.

## DISCUSSION

The main finding from the present study is that there are associations between higher ALT levels and some cardiometabolic risk factors including presence of MetS, high TC, high LDL, high TG and dyslipidemia.

A limited number of cohort studies have reported that NAFLD is an independent risk factor for incidence of CVDs.^1^^,^^28^ Since the prevalence of NAFLD is increasing among children and adolescents, it is important to investigate whether presence of NAFLD independently enhances the future risk of CVDs in this population.^29^

The relationship between liver enzyme levels and cardiometabolic risk factors has been extensively assessed in relation to adults. However, information regarding the distribution of liver enzymes and their association with cardiometabolic risk factors is scarce in relation to children and adolescents.^30^

ALT is a specific liver enzyme that relates to the cytosolic component of hepatocytes.^5^ On the other hand, other liver enzymes, including aspartate transaminase (AST), are also released from other organs such as the heart, skeletal muscles, kidneys, brain, pancreas and red blood cells.^6^ Liver enzyme levels are monitored routinely as part of clinical evaluations, and this form of assessment is accessible for many people.^31^

The use of ALT to screen for NAFLD is recommended in several national guidelines regarding overweight and obese children.^32^^,^^33^ According to thresholds derived from the National Health and Nutrition Examination Survey (NHANES), the sensitivities of ALT for detection of NAFLD are 72% for boys and 82% for girls; while the specificities are 79% for boys and 85% for girls.^34^

Some studies have demonstrated that undesirable changes in liver enzyme levels and liver fat content can be correlated with presence of diabetes mellitus and cardiovascular risk factors.^29^^,^^31^ However, the findings have been divergent and are thus incompatible in this regard. It is debatable whether a linear relationship exists between ALT levels and cardiovascular risk factors, given that some findings have demonstrated a U-shaped association between ALT levels and total mortality.^1^ However, some other studies have reported that ALT levels were associated with NAFLD, obesity, some features of MetS, fasting insulin levels and HOMA-IR as a marker for insulin resistance.^4^^,^^35^ One prospective study on Swedish men^36^ and another on Pima Indians^37^ found that high ALT levels, but not any other enzyme, including gamma-glutamyl transferase (GGT), was a risk factor for development of diabetes. Increasedliver enzyme activity may lead to inflammation that destroys insulin signaling. It has been shown that subjects with high ALT levels have higher levels of high-sensitivity C-reactive protein (hsCRP). HsCRP is an independent predictor for chronic disorders such as diabetes and cardiovascular disease.^38^

The Framingham Offspring Study demonstrated that enhanced ALT levels were related to an increase in risk factors for CVD after 20 years of follow-up, after adjusting for age and sex. However, this association disappeared with further adjustment.^5^

A positive correlation between the concentrations of some enzymes, including GGT, ALT, AST and ALP, and the risks of acquiring MetS and type 2 diabetes was shown in the Mexico City Diabetes Study.^39^ A study on Chinese patients with newly diagnosed type 2 diabetes reported that high ALT levels were strongly associated with some components of MetS and insulin resistance.^35^

A study on 1084 adolescents aged from 12.5 to 17.5 showed that presence of adverse cardiometabolic risk factors increased ALT and GGT levels and decreased the AST/ALT ratio in both genders. Overweight and obese adolescents had higher ALT and GGT levels and lower AST/ALT ratios than did leaner adolescents. There was an association between presence of liver biomarkers and central and overall adiposity in both genders. However, the correlations between presence of liver enzymes and the levels of blood pressure, insulin resistance and blood lipids were different between boys and girls. It was shown that ALT levels were correlated with higher overall cardiometabolic risk only in males. The AST/ALT ratio thresholds for determining that adolescents presented high cardiometabolic risk were 1.00 for younger males, 0.74 for older males, 0.86 for younger females and 0.87 for older females.^30^

Mohammadi etal. reported that there was a strong association between high levels of ALT, AST and ALT/AST ratio and most cardiometabolic risk factors. This relationship was independent of anthropometric indexes. These authors showed that high levels of liver enzymes can be considered to be a cardiometabolic risk factor during childhood.^40^

Studies that have investigated the association between NAFLD and CVD risk factors are scarce. However, one review article reported that presence of NAFLD is independently correlated with increased risk factors for CVD, according to a prospective study.^41^ A meta-analysis of population-based studies showed that there was a correlation between high GGT levels and increased risk factors for CVD. However, no correlation relating to ALT levels was found.^15^

We found that there was a significant association between ALT levels and BMI in linear regression models after adjusting for confounders. The coefficient of regression for waist circumference was borderline. Obesity is one of the risk factors for increased serum liver enzyme levels, particularly ALT, in both adults and children. An association between anthropometric measurements and biochemical complications, including increased ALT levels, was demonstrated among obese Japanese children.^38^

Measurement of ALT levels is commonly used in pediatrics. ALT measurement constitutes a readily available and low-cost blood test. It is used as a valuable screening test for detection of liver disease. However, the threshold value for high ALT levels and hence for diagnosing liver disease in pediatrics remains uncertain. In addition, the appropriate explanation for the results from ALT assays that are performed on children is unclear. Because of this indeterminacy, laboratories need to define what the normal range for ALT levels is, according to findings from their local populations.^34^

Clinicians need to be aware that high-normal ALT levels may be present in children with underlying chronic liver disease. Thereis evidence to suggest that lowering the cutoff for high ALT may improve sensitivity without a significant impact on specificity.^34^^,^^42^^,^^43^

The strength of the present study is its large sample size. Somelimitations that need to be acknowledged are its cross-sectional nature, lack of imaging procedures and observational design. Thus,we were unable to investigate causality. In addition, some potential confounders might have affected our findings.

## CONCLUSION

The present study showed that there were associations between higher ALT concentrations and the presence of MetS, high TC, high LDL, high TG and dyslipidemia. The findings suggest that a high level of ALT is beneficial for making pediatric clinical assessments on patients who may present high cardiometabolic risk. Clinicians who manage NAFLD patients should not focus on liver disease alone. They should also consider the increased chance of presentation of cardiometabolic risk factors.
